# MRI Safety for Interventional Radiologists: A Narrative Review for MRI-Guided Interventions

**DOI:** 10.7759/cureus.101084

**Published:** 2026-01-08

**Authors:** Pan Pan Ng, Pak Ki Yam, Wai Lun Poon

**Affiliations:** 1 Department of Diagnostic and Interventional Radiology, Queen Elizabeth Hospital, Hong Kong, HKG

**Keywords:** interventional radiology, magnetic resonance imaging (mri), mri-guided intervention, mri safety, tumor ablation

## Abstract

Magnetic resonance imaging-guided (MRI-guided) intervention has emerged as a rapidly growing field in interventional radiology centers worldwide, owing to its superior tissue contrast and ability to provide real-time imaging guidance without ionizing radiation. With the increasing adoption of MRI-guided intervention, it is important for interventionalists to understand the safety considerations for both staff and patients in the complex MRI and interventional environment. This article aims to provide an overview of the safety principles of MRI-guided interventions with reference to international guidelines and our local experience.

## Introduction and background

MRI-guided intervention involves the use of real-time magnetic resonance imaging to target and monitor minimally invasive interventional procedures. The application of MRI guidance in interventions provides excellent intrinsic soft-tissue contrast, enabling tumor localization and visualization of the ablation zone. It provides real-time imaging, which allows precise needle placement for both biopsy and ablation without exposing the patients to ionizing radiation. Since the first MRI-guided intervention in the 1990s, MRI systems have advanced continuously from low-field, open-bore systems to high-field, closed-bore systems with wide and short bore designs. Along with the increasing availability of MRI-compatible instruments, these advancements have enabled a wide range of interventions to be performed under MRI guidance. Current routine applications include breast [1], prostate biopsies [2], and ablation of renal [3,4], hepatic [5], prostate, and soft-tissue tumors [6,7], which have demonstrated clinical benefit.

Unlike other imaging modalities, MRI carries unique potential hazards related to three magnetic fields, each creating distinct risks: the strong static magnetic field exerts powerful attractive forces on ferromagnetic objects (the projectile effect); the radiofrequency (RF) field can induce electrical currents in conductive materials, causing burns and device heating; and the time-varying gradient field may induce peripheral nerve stimulation and is associated with acoustic noise. MRI-related safety incidents have been reported in approximately 0.05% of examinations, equating to one adverse event per 1,987 patients scanned [8]. Performing interventions in the MRI suites increases vulnerability to safety risks due to the larger number of personnel involved, increased diversity of instruments utilized, and prolonged interventional procedural time compared with a standard diagnostic MRI setting. Furthermore, patients undergoing interventional procedures are often sedated or anesthetized, increasing their susceptibility to thermal hazards [9]. These factors warrant additional safety precautions beyond those typically implemented in diagnostic MRI.

International MR safety guidelines have been established by multiple regulatory and professional organizations to guide safe MR practice. In recognition of the growing complexity of MR environments, specific recommendations addressing the safety of interventional MRI suites have been incorporated into the latest guidelines [10-12]. For example, the American College of Radiology (ACR) Manual on MR Safety advocates for augmented safety protocols with appropriate MR personnel training and zone-based access control for interventional MR suites [10]. Similarly, the United Kingdom’s Medicines and Healthcare Products Regulatory Agency (MHRA) Safety Guidelines for MRI Equipment in Clinical Use highlight the importance of MR safety risk reassessment for staff engaged in interventional work [11]. This review aims to synthesize the key safety principles in the context of MRI-guided interventions, focusing on projectile and RF-induced heating prevention, and the peri-procedural screening and monitoring, with reference to the international guidelines and insights from local experience.

## Review

Methods

This narrative review was conducted to synthesize current evidence on MRI safety considerations and preventive measures specific to MRI-guided interventions. A comprehensive literature search was performed using international databases, including PubMed, Embase, ScienceDirect, and Google Scholar, covering the period from Jan 1995 to August 2025. The search strategy combined keywords and Medical Subject Headings (MeSH) terms such as “MRI safety”, “MR guided interventional procedures”, “MRI safety” AND (“biopsy” OR “ablation”), “MRI” AND “thermal injury”, “MRI” AND “projectile injury”. Additional sources included manual reviews of reference lists from retrieved articles, international safety guidelines from the ACR, MHRA, and other relevant professional societies, as well as manufacturer technical documentation for interventional devices used in MRI-guided procedures. The inclusion and exclusion criteria were as follows.

Inclusion criteria: (1) English-language publications; (2) studies, case reports, or technical reports addressing MRI safety in the context of MRI-guided interventional procedures; (3) professional society guidelines and consensus statements on MRI safety; and (4) publications describing MRI safety hazards or preventive measures.

Exclusion criteria: (1) conference abstracts without full-text availability and (2) animal studies without clinical relevance.

Two authors (PN and PY) independently screened titles and abstracts for relevance, and full-text articles were subsequently retrieved. Discrepancies were resolved through discussion and consensus. No formal quality or risk-of-bias assessment tool was applied owing to the heterogeneity of study designs. The evidence was synthesized qualitatively and organized thematically according to the three primary MRI safety hazards and their associated preventive measures in the setting of MRI-guided interventions. Local experience, including institutional safety checklists and procedural workflows, was incorporated to provide practical context and implementation examples.

MRI safety hazards

Static Magnetic Field Hazards

Modern MRI scanners use superconducting magnets; the magnetic field is always “on” and extends beyond the scanner itself. The commonly used 1.5T (Tesla) MRI systems for interventional procedures generate fields approximately 30,000 times stronger than the Earth’s magnetic field. Within such strong fields, ferromagnetic objects are subject to both translational and rotational forces. Translational force is maximal at the opening of the magnet bore due to the high fringe field spatial gradient.

Projectile incidents have been reported to account for 4.8%-9% of all MRI adverse events [8,13]. Patient transport and mobility equipment, such as wheelchairs and stretchers, are the most common projectile objects, involving 26% of the projectile events in the 10-year review by the US Food and Drug Administration (FDA) [13]. Gas cylinders are also common projectile objects involved in reported incidents [13,14]; notably, a fatal incident in 2001, involving an oxygen cylinder that struck a pediatric patient, remains one of the most widely reported projectile-related deaths [15]. Similarly, common surgical instruments such as forceps and needles can behave as missiles and boomerangs under the strong magnet.

In addition, medical implants and devices with ferromagnetic parts may displace or rotate, resulting in tissue injury or device malfunction and rendering patients unsuitable for MRI-guided interventions. Reported incidents include fatal cerebral aneurysm clip displacement [16], pacemaker dysfunction and arrhythmia [17-19], and cochlear implant magnet migration [20,21]. A recent systematic review found magnetic dislodgement or migration was the most common MRI-related cochlear implant complication, accounting for 73% of the cases [20]. 

RF Heating and Burns

RF magnetic fields are responsible for the excitation of magnetic spins to generate MR signals for imaging. The B1 field can induce eddy currents in conductive materials (e.g., the human body and metals) within the bore during MRI scanning [22,23]. These currents may lead to heat generation and RF burns, particularly in areas of small skin contact where electrical resistance is the highest.

RF burns are the most frequently reported MRI adverse events, accounting for 32% of cases in a 10-year FDA review [13]. A recent systematic review estimated that 97% of thermal injuries could be prevented through proper safety protocols [24]. Common causes of RF burns include direct skin contact with the scanner, coil, or conductive material (most commonly ECG leads or wire loops), as well as direct skin-to-skin contact forming conductive loops. In recent years, novel fabric materials containing metallic microfibers (used in undershirts and sportswear) and face masks containing metal nose pieces or metallic antimicrobial coatings have been implicated in burn injuries following MRI examinations [25-27]. During the COVID-19 pandemic, incidents of facial burns from metal-containing face masks worn during MRI prompted the FDA to issue a safety communication regarding this RF hazard [28].

RF energy deposition can lead to increased body temperature and systemic overheating, especially in vulnerable populations, including pediatric patients, febrile individuals, and those under sedation or general anesthesia [9]. Energy deposition is quantified using the specific absorption rate (SAR). Factors contributing to high SAR include large body habitus and prolonged or repeated scanning with high SAR sequences [29].

Unlike diagnostic MRI examinations, in which only the patient is exposed to RF pulses, interventionalists may also face RF-related risks during MRI-guided procedures. For instance, during real-time imaging for needle insertion in an MRI-guided biopsy or ablation, the interventional radiologist’s arm or upper torso may extend into the bore to access the body part of the patient at the isocenter of the magnetic field. Moreover, sequences frequently used for needle position confirmation in MRI-guided interventions (e.g., BLADE, periodically rotated overlapping parallel lines with enhanced reconstruction) are typically high SAR sequences; therefore, repeated scanning during complex procedures may result in SAR values exceeding safety thresholds, leading to patient overheating and device heating. An in vitro study on various MR-conditional needles used for MRI-guided interventions demonstrated needle heating beyond physiological temperatures during prolonged MRI scanning [30]. 

Time-Varying Gradient Field Effects

Time-varying gradients are the rapidly applied gradients during scanning for slice selection and spatial encoding. The rapid switching of gradients can induce electric currents in the body, leading to peripheral nerve stimulation (PNS), involuntary muscle contraction, and pain. PNS is infrequently reported, and most patient experiences are mild [31]. This typically occurs in sequences with high amplitude, rapidly switching gradients, such as echo planar imaging, which includes diffusion-weighted imaging (DWI) used in MRI-guided prostate biopsy. Addressing the safety issue of interventionalists working in close proximity to the MRI bore, the study demonstrated a substantially higher PNS threshold above patient safety limits in different arm positions using human models [32].

The switching gradient coils also produce vibration against their mountings, generating the characteristic loud acoustic noise of MRI, which can result in temporary or permanent hearing damage [33,34] (Table 1). The noise level can exceed the International Electrotechnical Commission (IEC) exposure limit of 99 dBA, necessitating hearing protection [35,36]. Effective hearing protection is critical for interventionalists who are frequently exposed to loud noise during real-time needle placement in MRI-guided interventions. Recent research has demonstrated the safety of cumulative noise exposure to participants undergoing multiple MRI studies, provided that adequate hearing protection was used [37].

**Table 1:** Summary of reported MRI safety incidents and their causes

Hazard category	Incidents description
Projectile hazards	Oxygen cylinder turned into missile resulting in fatal head injury of a paediatric patient [15]
	Patient transported into scan room on ferromagnetic wheelchair and thrown into the magnet resulting in lower limb fractures [13]
Implant malfunction	Ferromagnetic cerebral aneurysmal clip torqued in magnetic field resulting in fatal intracranial haemorrhage [16]
	Pacemaker-dependent patient with pacemaker dysfunction after MR study requiring replacement of pacemaker generator [19]
	Cochlear implant magnet migration and dislocation associated with MRI [20]
RF heating	Third degree burns from ECG-leads in an anesthetized patient underwent MRI study [13]
	Second degree burns due to inadequate padding between coil cable and skin [13]
	Patient suffered burn injury from jogging pants [25]
	Patient suffered permanent neurological damage due to RF heating associated with deep brain stimulation leads [38]
Acoustic noise	Young patient developed bilateral sensorineural hearing loss after 1.5T MRI scan without hearing protection [33]

Operational safety measures

Zone Access Control 

**Figure 1:**
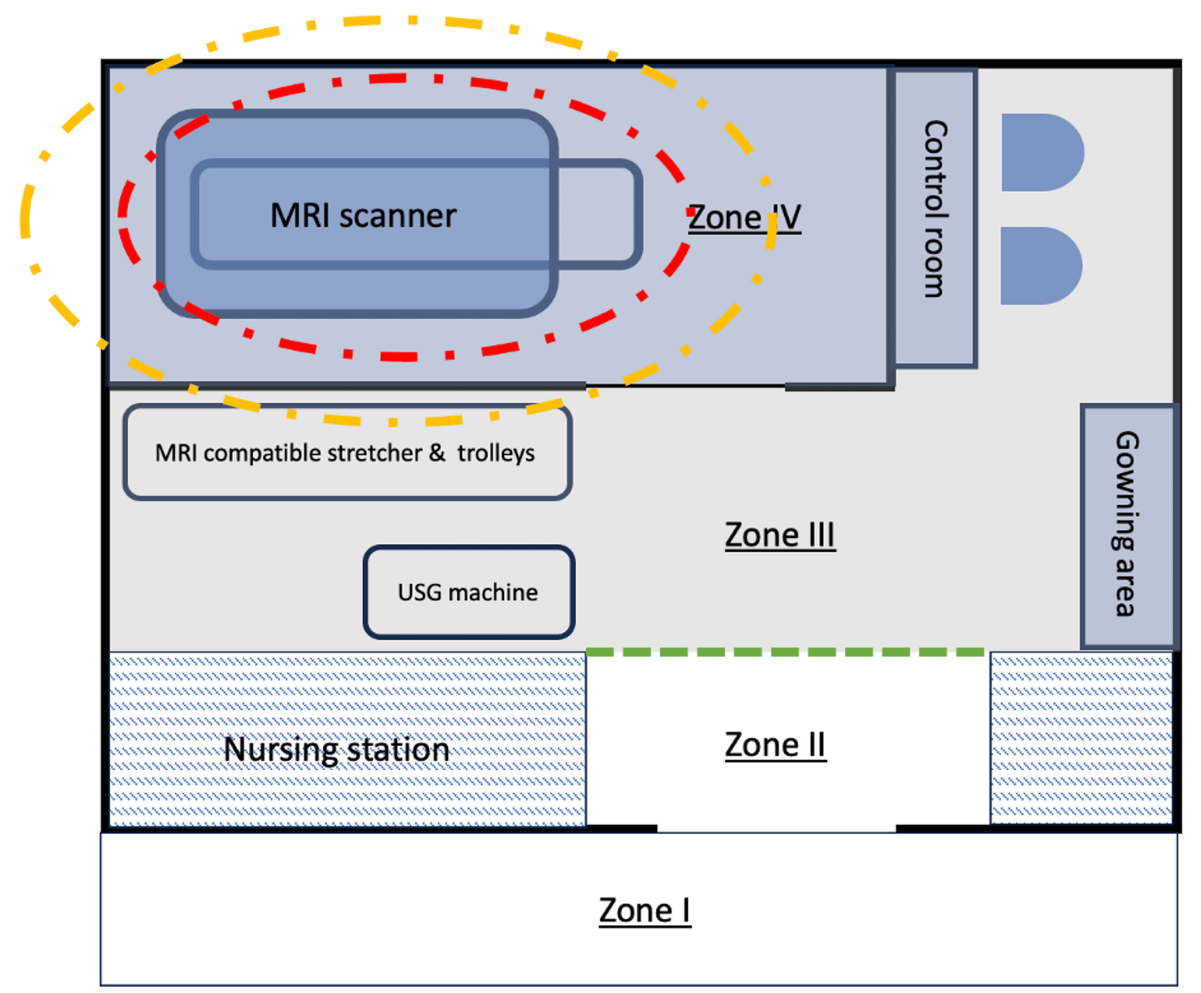
Floor plan of the interventional MRI suite in our center Image created by the authors using the tool PowerPoint. It illustrates the zoning of the intervention MRI suite in our institute. The MRI projectile area (red dash line) is contained within Zone IV, while the 9-gauss line may extend beyond Zone IV into Zone III (yellow dash line). MR-compatible stretcher and trolleys for patient and instruments transfer into zone IV are parked in Zone III. The MR-unsafe ultrasound (USG) machine is parked in Zone III away from Zone IV. At the entrance of the restricted Zone III, line and warnings are demarcated on the floor to help access control (green dash line).

**Figure 2:**
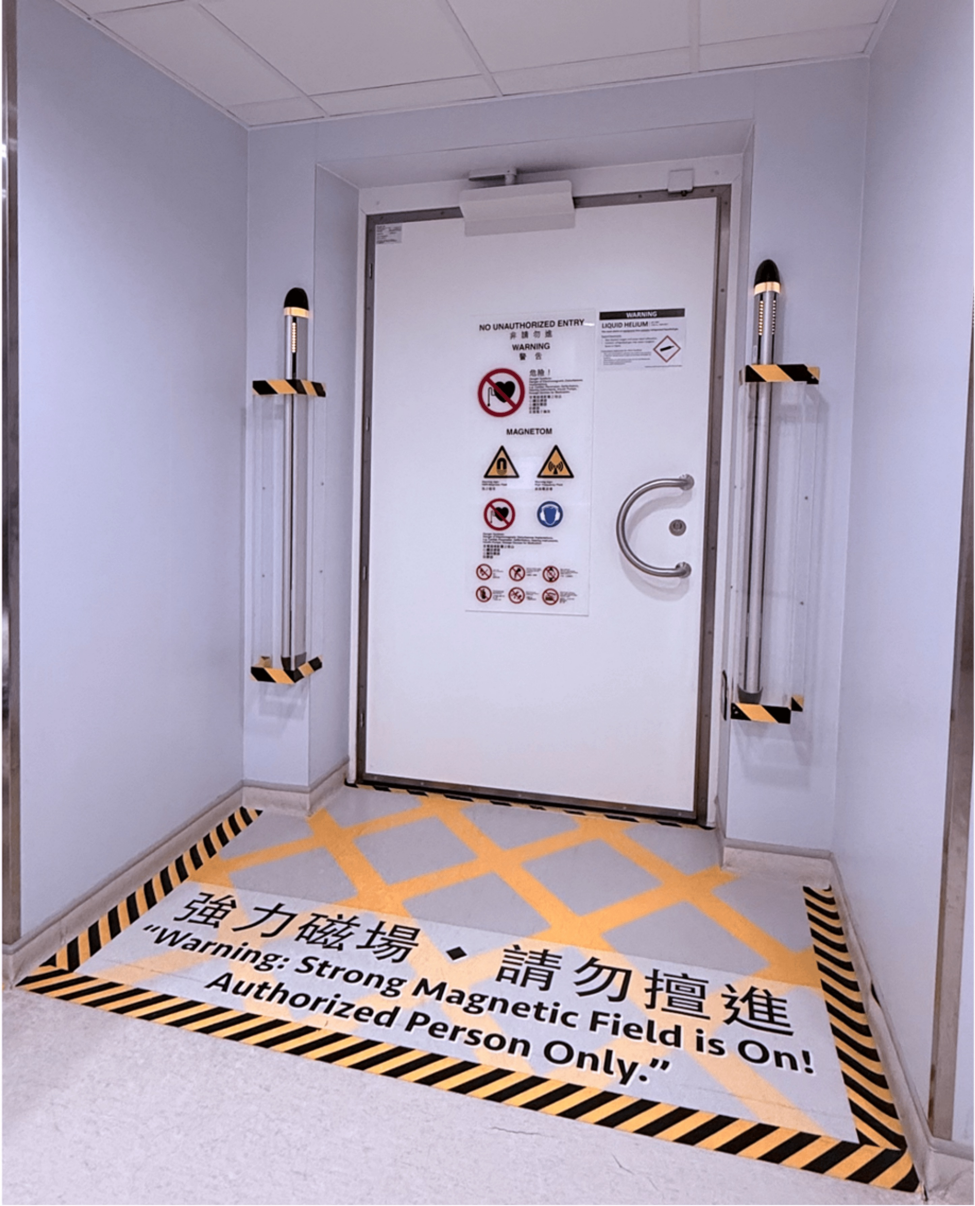
Entrance to Zone IV (MR scanner room) Photograph showing the warnings outside the MR scanner room, stating the presence of a strong magnetic field. The door of the scanner room should remain closed, except for patient care. Ferroguard, the ferromagnetic detection system, is installed at the entrance to serve as one of the MR safety measures.

To mitigate hazards arising from the static magnetic field, the ACR recommends dividing MRI facilities into four conceptual zones, with corresponding levels of access control (Table 2) [10]. All patients or non-MR personnel entering Zone III must undergo screening for ferromagnetic objects and implants, as the magnetic fringe field can be sufficient (i.e., the 9-gauss line) to pose hazardous effects on cardiac implantable electronic devices (CIEDs). Zone IV contains the MR projectile area where unscreened ferromagnetic objects can result in severe injuries (Figures 1-2).

**Table 2:** MRI safety zones recommended by the ACR manual on MRI safety ACR: American College of Radiology

Zones	Description of the zones
Zone I	Freely accessible to the general public.
Zone II	Interface between the uncontrolled Zone I and the MR-controlled access area. Contains patient waiting and changing area, nursing area. Patient screening typically takes place in Zone II.
Zone III	Within the MR-controlled access area. Restricted access due to fringe field risk.
Zone IV	Equivalent to the MR scanner room. Within the MR-controlled access area and containing the MR projectile area. Strict controlled access, doors remain closed, except for patient transfer.

In practice, the projectile risk zone of individual ferromagnetic objects varies. Field strength below 30 gauss generally poses little projectile risk for most ferromagnetic objects [12]. Most MR-conditional equipment commonly used in Zone IV, including anesthetic machines and monitoring equipment, functions safely at maximum field strengths of 200-300 gauss [39,40]. Currently, there are no international recommendations mandating floor marking of iso-gauss lines; however, it is considered a useful practice for interventional suites to demarcate iso-gauss lines on the floor for ease of reference [10,12,41,42]. The 9-, 30-, 200-, and 300-gauss lines are the common thresholds implemented.

Access control should be maintained at all times, including during emergency situations. To prevent MR hazards during crises, a designated resuscitation area should be established in a magnetically safe location, outside Zone IV and beyond the 9-gauss line, usually in Zone III or II. Routine MR safety labeling of equipment should be maintained [11]. In the event of a medical emergency, the priority is to immediately transfer the patient with an MR conditional stretcher from Zone IV to the designated resuscitation area while initiating basic life support [10]. Evacuation routes must remain unobstructed, and the scanner room door should be closed after patient extraction to prevent emergency responders from entering with ferromagnetic equipment. MRI personnel should serve as gatekeepers, communicating Zone IV hazards to emergency responders and non-MR personnel. Any emergency entry of unscreened personnel or unlabeled equipment to the scanner room should be prohibited. Multidisciplinary team training and regular evacuation drills are essential to ensure operational efficiency [43].

MRI Safety of Interventional Devices 

**Figure 3:**
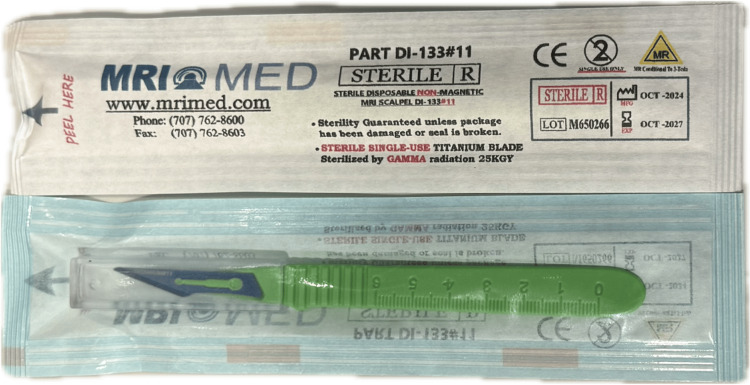
MR-conditional titanium scalpel (MRI Med, Petaluma, CA)

Apart from the MR conditional physiological monitor, ECG electrodes, anesthetic machine, and infusion pump, which are familiar in diagnostic studies, MRI-guided interventions typically require a wide range of devices that are uncommon for diagnostic MRI studies. Many of the standard surgical or interventional instruments are not applicable to an MR environment, and MR-compatible alternatives should be sourced. For example, ceramic or titanium alloy scalpels are the safe and functional alternatives to the strongly ferromagnetic martensitic stainless-steel scalpels (Figure 3). Titanium, inconel, nitinol, and plastic polymers are common materials used in MR-compatible biopsy needles, co-axial and ablation needles, given their mechanical strength and MR visualization properties [44-46]. Hypodermic needles used for the administration of local anesthesia are made of austenitic stainless steel, which exhibits very low ferromagnetic properties in the 1.5T system [47]. In our experience, standard hypodermic needles experienced a very weak attractive force when used near the bore. To minimize the projectile risk, the needles should be used at a distance away from the opening of the MRI bore.

The American Society of Testing and Materials (ASTM) International Committee established a labeling system that categorizes device MRI safety into three types (ASTM F2503) [48] (Table 3). It is recommended that only MR safe or MR conditional devices be allowed in Zone IV. Prior to the MRI-guided procedures, careful planning is necessary to determine the safety of each procedural step with respect to both the location of intervention and the MRI compatibility of the instruments involved. For example, procedural steps requiring ultrasound (USG) guidance should ideally be performed in Zone III prior to transferring the patient into Zone IV, as USG machines are considered MR unsafe. However, this may not always be practical. If portable metallic or partially metallic instruments that are MRI unsafe or lack MR safety labeling are deemed essential for patient care within Zone IV, their introduction and safe locations in Zone IV should be extensively tested by qualified experts using standard methodologies [49-52]. In addition, proper tethering should be employed to mitigate projectile risks [10]. For portable metallic or partially metallic instruments that are designated as MR conditional, such as monitors and anesthetic machines, their functional locations within Zone IV should be clearly defined based on specified maximum field strength limits, typically identified by iso-gauss lines or designated markings on the floor. 

**Table 3:** ASTM MRI safety labelling system ASTM: American Society of Testing and Materials

Category	Description
MR safe	No known hazards in any MR environment. Non-magnetic, non-metallic, electrically non-conductive materials. Examples: plastics, ceramics, and glass.
MR conditional	Safe under the specified MR conditions. Specifications: static magnetic field strength, spatial field gradient, RF exposure, scanner type, orientation, and slew rate.
MR unsafe	Unacceptable risks in all MR environments.

Pre-procedural Screening and Safety Check

Pre-procedural screening is one of the most important steps to ensure safety and should occur on multiple occasions and by qualified personnel. The ACR manual on MR safety suggests at least two separate screenings before access of any patients or non-MR personnel into the MRI scanner room [10].

A first-level patient screening should be conducted when the examination or procedure is requested. In our institution, patients are interviewed by interventional radiologists in the clinic, who check for contraindications to MRI-guided interventions, including foreign bodies, implants, and claustrophobia. For active medical implants, such as CIEDs and programmable ventricular shunts, specific pre- and post-procedure interrogation and programming should be arranged in advance.

A second-level screening occurs when patients arrive at the MRI suite. Patient screening should be carried out in Zone II by MR personnel with the aid of standardized questionnaires, inspection, and ferromagnetic detection systems, similar to those of a diagnostic MRI examination. The results of screening should be clearly documented in the hospital records (Table 4).

**Table 4:** QEH MRI checklist Local MRI safety screening checklist for inpatient and patient under general anaesthesia and MRI-guided procedures.

Item	Yes	No
MRI non-compatible/unsafe oxylog/oximeter	☐ Remove ☐ Change to compatible	
MRI non-compatible/unsafe infusion device/syringe pump	☐ Remove ☐ Change to compatible	
MRI non-compatible/unsafe oxygen cylinder	☐ Remove ☐ Change to compatible	
Peep valve with metal parts	☐ Remove ☐ Change to compatible	
Metallic mouth prop	☐ Remove ☐ Change to compatible	
Oxygen mask with metal parts	☐ Remove ☐ Change to compatible	
MRI non-compatible/unsafe ECG electrodes/leads/defibrillator pads	☐ Remove ☐ Change to compatible	
MRI non-compatible/unsafe pressure bag	☐ Remove ☐ Change to compatible	
MRI non-compatible/unsafe metallic neck collar/halo ring/splint/external fixator	☐ Remove ☐ Change to compatible	
Drains with metallic artery forceps	☐ Remove ☐ Change to compatible	
Metallic hanger	☐ Remove ☐ Change to compatible	
Medication patches	☐ Remove ☐ Change to compatible	
Patient’s card label	Remarks: Checked by: ________________ (DIR Nurse) Checked by: ______________ (Radiographer) Date: ___________________	

In addition to patient screening, staff, including interventionalists, scrub nurses, and anaesthetists, should be screened and checked for ferromagnetic objects or implants before being allowed to enter the MR environment. For interventionalists or nurses whose arms may be scanned and exposed to the RF energy in the MR bore, precautions should also be taken against RF-related hazards. Any clothing with metallic fibers or accessories should not be worn underneath the surgical gown, and personnel should preferably change into pocketless MR-safe gowns. Extensive tattoos on the arm may require prophylactic cold compresses.

For implants and interventional instruments, a systematic approach should be used to validate their MR safety status. The primary method of validation is to obtain information directly from the device manufacturer, including product labeling, instructions for use, ASTM classification on MR safety (MR safe, MR conditional, or MR unsafe), and any specified conditions for MR conditional devices. The specifications typically include the permitted static field strength, spatial field gradient, gradient slew rate, SAR limits, and any essential device setting or positioning [40]. When manufacturer information is not available or ambiguous, validated MR safety databases can be useful in providing peer-reviewed safety testing data and scanning parameters for implants and devices, including reference manuals, MRIsafety.com, or institutional MR safety databases if available [53,54]. Institutional safety verification protocols should be established to designate MR personnel responsible for the verification, to define the acceptable sources of safety information, outline procedures for handling devices with unknown safety information, and document the verification process.

According to the latest ACR recommendation [10], an additional augmented full stop and final check should be performed by a Level 2 MR technologist together with a second MR personnel, for the patient, supporting equipment, and staff, before entering Zone IV for complex MR environments, including MR interventions. This is particularly important for procedures that lead to changes in patient or equipment status in Zone III. An illustrative example is a combined USG- and MRI-guided tumor cryoablation, where we perform USG-guided cryoprobe placement in Zone III and transfer the patient into Zone IV for MR guidance. This leads to a change in patient status that differs from the initial screened status in Zone II. A final check before moving the patient into Zone IV is essential to ensure that any MR-unsafe needles or equipment utilized in Zone III are removed and not hidden within the surgical drapes or around the patient.

Intra-procedural Monitoring and Safety Measures

**Figure 4:**
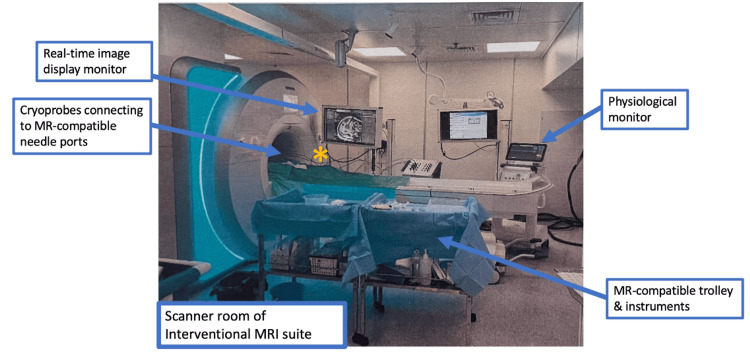
MR scanner room of the interventional MRI suite in our institution Photograph showing the room layout during MRI-guided cryoablation of a renal tumor. The portable MR-compatible real-time image display monitor is placed near the scanner for optimal visualization by the interventionalists. The cryoablation system and procedural trolleys were in their designed positions to maximize workflow and safety. Continuous physiological monitor is available inside the scanner room allowing real-time assessment throughout the intervention. Special care is taken to arrange and pad the ablation probe cables, preventing direct contact with patient’s and interventionalist’s to minimise risk of thermal injury, as indicated by the asterisk*.

Before scanning begins, appropriate patient positioning should be established to prevent skin-to-skin contact and formation of conductive loops. Padding should be applied to avoid skin contact with the magnet bore or cables, and between limbs to protect the patient from RF burns. During the intervention, any changes in patient positioning or introduction of new cables (e.g., from ablation probes) should be properly arranged and padded against conductive loops and skin contact before resuming scanning (Figure 4).

Continuous physiological monitoring of vital signs is essential during MRI-guided procedures. Attention should also be given to signs and symptoms of overheating, especially for vulnerable or sedated patients. To limit systemic heating, the International Electrotechnical Commission (IEC) standard restricts scanning for patients with compromised thermoregulation or patients unable to communicate pain to Normal Operating Mode, with a whole body SAR limit of 2.0W/kg and ≤0.5°C core temperature rise [35]. For device heating, interventional devices are tested according to ASTM F2182, with the majority of their labeling limited to Normal Operating Mode or with a specific B1+rms threshold (root-mean-square of B1+ field). First-level Controlled Mode is generally contraindicated for interventional devices due to higher RF power.

**Figure 5:**
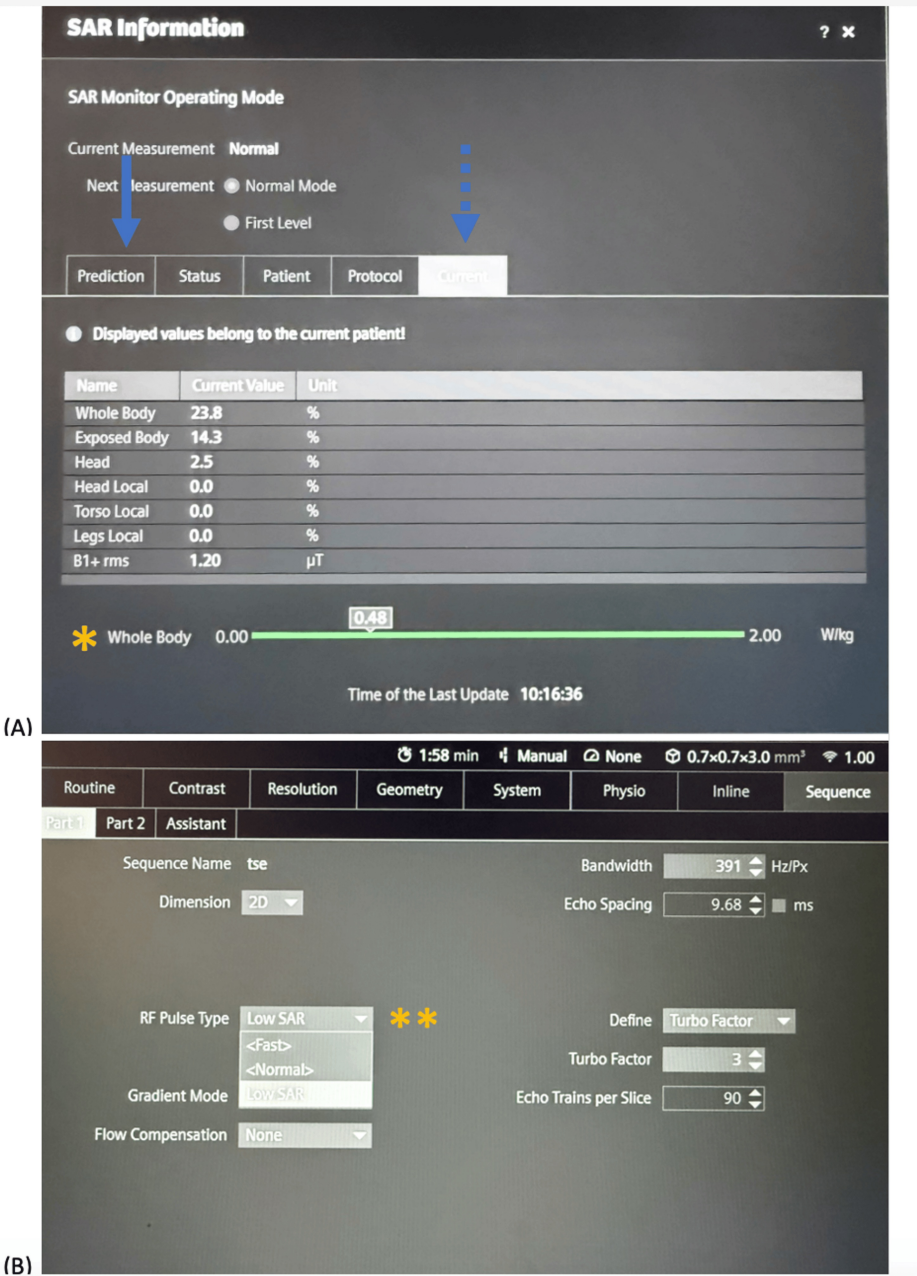
Specific absorption rate (SAR) monitoring during MRI-guided intervention (A) Photograph showing the software interface of a Siemens Aera 1.5T MR scanner. The software displays the predicted SAR value for a planned sequence (arrow). Real-time whole body SAR values are also available for intra-procedural monitoring (dash arrow and asterisk*). (B) Photograph showing manufacturer-provided options for RF pulse modification, including a low SAR module (asterisk**), to reduce the SAR exposure when approaching operational limits.

MR technologists should monitor and minimize the RF exposure, as repeated or prolonged scanning may be needed during the intervention to confirm needle position and monitor the ablation zone. Pre-sequence prediction and real-time SAR and B1+rms are available from the scanner software (Figure 5). The operators will be alerted when SAR approaches or exceeds the limits for the corresponding mode of operation. Under these circumstances, MR technologists should modify the scan parameters, for example, by reducing the flip angle and minimizing the number of slices while balancing image quality. When necessary and appropriate, cooling periods should be implemented to allow dissipation of RF energy. Other MR temperature monitoring techniques include fiber-optic-based temperature sensors used for ablation needles and catheters, and non-invasive MR thermometry that can provide real-time thermal mapping of tissue [55,56]. 

Safety Policies and Staff 

In view of the complexities of interventional MRI facilities, a local MRI safety committee should be established to develop, implement, and review intervention-specific safety policies and standard operating procedures. The committee should include multidisciplinary experts with clearly defined roles and responsibilities to address different aspects of safety management. For instance, each MRI facility should be overseen by an MR Medical Director (MRMD), while safety measures should be implemented and enforced by the MR Safety Officer (MRSO). An MR Safety Expert (MRSE) should be available to provide advisory support [10]. The committee is responsible for credentialing and training all personnel authorized to work in the MR environment and for maintaining corresponding documentation. Regular reviews and updates should be conducted, particularly when new procedures or interventional devices are introduced [28]. All safety incidents and near-misses should be reported to and investigated by the committee to facilitate continuous improvement. 

Future directions

Current MR safety recommendations for MRI-guided interventions remain largely extrapolated from the well-established diagnostic MRI literature, with supplementary consideration of specific risk factors inherent to the interventional environment. Large-scale and prospective analyses specifically addressing safety in MR-guided interventions are lacking in the peer-reviewed literature. As the volume of MRI-guided interventions continues to expand, the development of dedicated safety registries and guidelines specific to MRI-guided interventions is necessary for data analysis and standardization of safe practice.

Emerging advancements of MRI robotic systems demonstrate potential to improve procedural efficiency and reduce patient and occupational MR exposure in MRI-guided interventions. Fully automated or assisted needle insertion systems can reduce procedural time through automated needle realignment guidance, thereby minimizing the need for needle repositioning and overall magnet time [57,58]. While initial data are promising, prospective comparative studies in clinical procedures remain limited at this early stage.

The Integration of artificial intelligence (AI) algorithms into MRI-guided intervention workflows offers multiple mechanisms to enhance patient safety. For example, AI-based systems have demonstrated high accuracy in implant detection from chest radiographs [59]. AI algorithms can provide patient and treatment-specific SAR prediction and generate real-time SAR maps [60]. Digital Twin models that mirror patient and device characteristics offer the potential to simulate device heating and artifacts before the actual procedure, which can be valuable for novel device testing and new procedures [61]. Clinical implementation of these AI-based techniques requires further validation and maturation.

## Conclusions

MRI-guided interventions provide superior, real-time image guidance while eliminating exposure to ionizing radiation for both the patients and staff. With continued advances in MRI technology and interventional techniques, the range of MRI-guided procedures will inevitably expand, accompanied by the increasing complexity of the MRI environment.

A comprehensive understanding of the fundamentals of MR safety hazards, proper device selection, meticulous screening and safety checks, comprehensive training, and application of standardized safety protocols form the cornerstones of safe and effective MR practice. Achieving excellence in MRI-guided interventions requires not only technical expertise in interventional procedures but also specialized knowledge of MR safety considerations, as well as the collective commitment of multidisciplinary personnel.
